# PSMA PET-directed radiotherapy for prostate cancer: From precision planning to future innovations

**DOI:** 10.1002/VIW.20250143

**Published:** 2025-10-31

**Authors:** Wenpeng Huang, Jessica C. Hsu, Ruobing Li, Kexin Lan, Xin Qi, Yuchun Wei, Weibo Cai, Hongzhen Li

**Affiliations:** 1 Department of Nuclear Medicine, Peking University First Hospital, Beijing, China; 2 Departments of Radiology and Medical Physics, University of Wisconsin – Madison, Madison, Wisconsin, USA; 3 School of Health Sciences, Purdue University, West Lafayette, Indiana, USA; 4 College of Letters and Science, University of Wisconsin – Madison, Madison, Wisconsin, USA; 5 Department of Radiation Oncology, Peking University First Hospital, Beijing, China; 6 Department of Radiation Oncology, Shandong Cancer Hospital and Institute, Shandong First Medical University and Shandong Academy of Medical Sciences, Jinan, Shandong Province, China

**Keywords:** biology-guided radiotherapy, metastatic disease, personalized medicine, PET/CT, prostate cancer, PSMA, radiation therapy

## Abstract

Positron emission tomography (PET), particularly with prostate-specific membrane antigen (PSMA) tracers, has revolutionized the clinical management of prostate cancer (PCa). This review highlights the pivotal role of PET molecular imaging in guiding radiotherapy (RT) across diverse clinical scenarios, from postoperative biochemical recurrence to oligometastatic disease. Growing evidence shows that PET excels in lesion detection, enhances target volume delineation, enables focal dose escalation, and guides treatment intensification. PSMA PET increases the precision of RT planning, supports personalized therapeutic approaches, and is associated with improved outcomes, including biochemical recurrence-free and metastasis-free survival. The integration of PET with advanced RT technologies, including biology-guided radiotherapy (BgRT), is paving the way for real-time, biologically adaptive treatment paradigms. However, challenges remain, including the need for standardized protocols, management of tracer variability, and clinical translation of innovations such as PET-linear accelerator (LINAC) into routine practice. Future research should prioritize large-scale, prospective studies to establish the clinical efficacy, cost-effectiveness, and optimal integration of PET-guided RT in PCa care.

## 1 | INTRODUCTION

Prostate cancer (PCa) is the second most commonly diagnosed cancer globally (37.5 per 100,000) and the most prevalent cancer among men,^1–3^ with incidence rates varying significantly across geographic regions.^4^ Radiation therapy (RT), which depends heavily on advanced imaging and computational techniques, delivers ionizing radiation precisely to tumor sites with the goal of eradicating cancer cells or inhibiting their growth while minimizing exposure to surrounding healthy tissues and vital organ.^5–8^ Modern RT modalities include intensity-modulated radiotherapy (IMRT), image-guided radiation therapy (IGRT), stereotactic body radiation therapy (SBRT), and biology-guided radiotherapy (BgRT).^2,9,10^

In PCa management, RT is applied at various stages of the disease, serving curative (radical), adjuvant, salvage, or palliative purposes.^11,12^ The integration of imaging technologies has been pivotal in advancing RT planning and delivery. Currently, computed tomography (CT) remains the standard imaging modality for treatment simulation in radiation oncology. However, CT primarily captures anatomical changes between treatment sessions and does not reflect dynamic biological processes within the tumor. As medical technology evolves, innovative imaging approaches are refining therapeutic strategies.^13^ Positron emission tomography (PET), a well-established molecular imaging technique, is increasingly utilized for lesion localization in PCa. By enabling visualization and quantification of tumor-specific biological features, PET has significantly improved the precision and accuracy of RT planning and delivery.^6^

**FIGURE 1:**
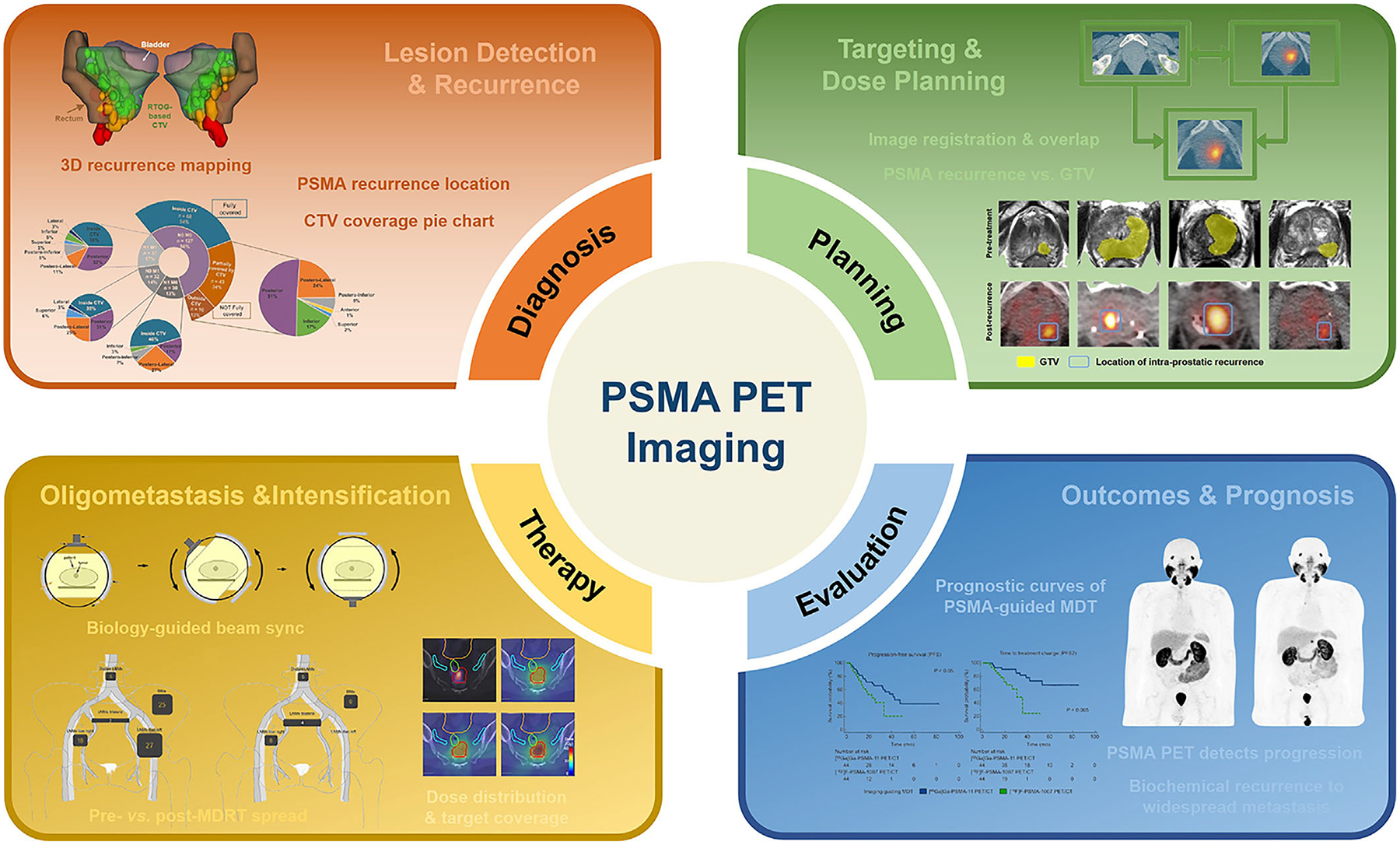
Schematic overview illustrating the integrative role of prostate-specific membrane antigen (PSMA) positron emission tomography (PET) imaging in prostate cancer management, spanning recurrence detection, target delineation, and outcome evaluation throughout the radiotherapy workflow.

In this comprehensive review, we first explore the interplay between PET molecular imaging and recent advances in RT for PCa. We then examine the pivotal role of PET in guiding RT planning and clinical decision-making, with particular emphasis on its applications in disease staging and individualized treatment strategies. Special attention is given to studies published within the past 3 years, focusing on PET’s effectiveness in detecting recurrent lesions in patients with postoperative biochemical recurrence, its precision in target delineation and dose adaptation, its role in stereotactic RT for oligometastatic disease, and its impact on long-term outcomes and prognostic assessment (Table 1). Finally, we discuss the significance of PET-based RT planning and the translational potential of emerging PET tracers and BgRT approaches, underscoring the need for future large-scale clinical trials to validate the clinical benefits of these advanced workflows in routine practice (Figure 1).

## 2 | PATHOGENESIS, CLASSIFICATION, AND RT OF PCA

### 2.1 | Pathogenesis and classification

The molecular landscape of PCa has been extensively characterized in recent years, revealing distinct biological pathways that drive tumor initiation, progression, and therapeutic resistance.^14^ At the genomic level, PCa exhibits substantial heterogeneity, with recurrent somatic alterations that include chromosomal rearrangements, most notably the androgen-regulated TMPRSS2–ERG fusion, which is present in approximately 50% of cases.^15^ Loss of tumor suppressor genes (PTEN, TP53, and RB1) and defects in DNA damage repair pathways, particularly involving BRCA1/2 and mismatch repair genes, also contribute to the pathogenesis of PCa.^16^ The androgen receptor (AR) signaling axis remains the central oncogenic driver throughout disease progression, making androgen-deprivation therapy (ADT) one of the standard treatments for PCa.^17,18^ However, the incidence of castration-resistant PCa (CRPC) has been rising, possibly due to clonal selection of rare preexisting AR-low/negative clones or transdifferentiation of AR-positive adenocarcinoma.^19,20^

PCa management relies on robust clinical grading and staging systems to guide treatment and predict outcomes. Histological classification is anchored by the Gleason scoring system, which evaluates tumor aggressiveness based on glandular architecture. In 2013, Pierorazio et al.^21^ refined this system by introducing Gleason Grade Groups, a five-tier prognostic classification based on specific Gleason scores. The International Society of Urological Pathology (ISUP) endorsed this system in 2014 to improve risk stratification, with evidence supporting its predictive value for biochemical progression-free survival (bPFS) in patients undergoing radical prostatectomy (RP) or RT.^22,23^ More recently, deep learning and artificial intelligence techniques have shown promise in enhancing the precision and reproducibility of pathological assessments.^24,25^ While a global consensus on PCa risk stratification has yet to be reached, nearly all clinical guidelines recommend combining Tumor–Node–Metastasis (TNM) staging, Gleason Grade Group, and prostate-specific antigen (PSA) level as the core parameters for risk classification.

Current clinical classification generally follows two complementary frameworks: anatomical disease extent and therapeutic response patterns. The TNM system, a globally standardized framework for solid tumor staging, categorizes PCa into localized disease (T1-T2, N0, M0), locally advanced disease (T3-T4, N0, M0), lymph node-positive disease (Any T, N1, M0), and metastatic disease (Any T, Any N, M1). Anatomical staging is traditionally based on conventional imaging modalities such as CT and magnetic resonance imaging (MRI). Precision has improved with the integration of advanced imaging techniques, including PET. Meanwhile, treatment response-based classification defines two major subgroups: hormone-sensitive prostate cancer (HSPC), which responds to ADT, and CRPC, characterized by disease progression despite castrate-level testosterone (<50 ng/dL). With the clinical burden of CRPC increasing, recent therapeutic advances have focused on intensifying treatment in the HSPC setting to delay progression toward CRPC. Examples include the combination of novel AR pathway inhibitors (ARPIs) with traditional ADT.^26^

### 2.2 | PET in the diagnostic evaluation of PCa

PET has revolutionized the diagnosis and staging of PCa, particularly in the challenging context of biochemical recurrence. Traditional fluorodeoxyglucose (FDG) PET is less effective for PCa due to the tumor’s low glycolytic activity and interference from urinary excretion. However, the development of advanced radiotracers has addressed many of these limitations. Radiolabeled choline PET/CT (e.g., [^18^F]-choline) enhances detection of lymph node and bone metastases in biochemical recurrence (BCR) patients with elevated PSA levels, although its sensitivity declines at lower PSA values and specificity for malignancy remains suboptimal. The amino acid-based tracer [^18^F]-fluciclovine was developed to overcome some of these drawbacks and has demonstrated superior performance compared with choline PET, particularly for evaluating the prostate bed and in patients with low PSA levels. Its efficacy led to US FDA approval for use in BCR. The most transformative advance, however, has been the clinical adoption of prostate-specific membrane antigen (PSMA)-targeted PET radiotracers, such as [^68^Ga]Ga-PSMA-11, [^18^F]-DCFPyL, and [^18^F]-PSMA-1007. PSMA is a transmembrane glycoprotein with relatively selective overexpression in prostatic adenocarcinoma, enabling exceptionally high tumor-to-background contrast and establishing PSMA PET as a cornerstone modality in PCa management.^27^

Although initially applied in recurrent PCa, PSMA PET has shown increasing value in primary disease. In 2016, Fendler et al.^28^ systematically evaluated [^68^Ga]Ga-PSMA-HBED-CC PET/CT and demonstrated that a SUVmax threshold ≥6.5 strongly predicted malignancy, achieving a positive predictive value (PPV) of 95%. Subsequent studies^29–35^ have consistently confirmed the high diagnostic accuracy of PSMA PET compared with conventional imaging modalities, while also underscoring its complementary role alongside multiparametric MRI (mpMRI) in primary PCa, supporting lesion detection, biopsy guidance, and initial risk stratification. In recurrent PCa, PSMA PET exhibits even greater efficacy and is considered the imaging modality of choice for patients with BCR. Current evidence demonstrates that PSMA PET achieves significantly higher detection rates than conventional imaging, with sensitivity ranging from 75% to 97% and specificity between 80% and 100%, even at remarkably low PSA levels of 0.2–0.5 ng/mL.^36–38^

Importantly, PSMA PET provides highly accurate lymph node staging prior to dissection^39,40^ and can detect bone metastases that may be equivocal on CT, MRI, or bone scintigraphy.^41–44^ This superior diagnostic performance has established PSMA PET as the standard imaging modality for PCa staging, with studies reporting that it upstages 15%–30% of newly diagnosed intermediate-to-very-high-risk PCa cases by detecting lymph node or distant metastases missed by conventional imaging.^45^ PSMA PET is also widely utilized for restaging in recurrent or persistent disease following curative-intent treatment,^46^ where accurate assessment of extra-prostatic spread is critical for treatment success.

Beyond tumor detection and staging, PSMA PET enables precise delineation of disease burden, allowing for more personalized local and systemic therapy. In the setting of BCR, PSMA PET can guide salvage radiotherapy (sRT) by pinpointing the site of recurrence, or lead to escalation to systemic therapy if distant metastases are found.^47^ Furthermore, PSMA-PET is adept at identifying oligometastatic disease that is often missed by conventional imaging. This intermediate disease state, between localized and widespread metastatic cancer, supports a paradigm shift toward metastasis-directed therapy (MDT), such as salvage lymph node dissection or stereotactic ablative radiotherapy (SABR), which may delay systemic therapy and potentially improve outcomes.^48^ In addition, PSMA PET is indispensable for selecting appropriate candidates for radionuclide therapy. The degree and distribution of PSMA expression on PET directly predict patient eligibility and potential response to PSMA-targeted radioligand therapies, such as [^177^Lu]Lu-PSMA-617.^49–51^

### 2.3 | Radiotherapy in PCa

Modern radiotherapy (RT) for PCa includes external beam radiotherapy (EBRT), brachytherapy, and proton therapy. The foundation of EBRT lies in IMRT and its rotational variant, volumetric-modulated arc therapy (VMAT/RapidArc). These techniques use dynamic multileaf collimators to sculpt radiation doses precisely to the prostate’s irregular shape, reducing rectal dose by 30%–50% and bladder dose by 40% compared to older 3D-conformal RT techniques. IMRT and VMAT are now considered standard of care across all risk categories. SBRT delivers ultra-hypofractionated regimens using advanced image guidance and motion management. It has demonstrated noninferior biochemical control with low toxicity in low-to-intermediate-risk disease, while also allowing targeted dose escalation to areas at risk for microscopic tumor involvement that are not detectable on conventional imaging (i.e., suspected subclinical regions).^52–54^

Brachytherapy delivers radiation directly to the tumor area via implanted radioactive sources. Low-dose-rate brachytherapy is an excellent monotherapy option for low-risk disease. For intermediate-to-high-risk diseases, it serves as a boost following EBRT, providing superior survival benefits compared with dose-escalated EBRT alone.^55^ High-dose-rate brachytherapy allows for optimized dose painting and is commonly used as a boost with EBRT for high-risk disease or as monotherapy for intermediate-risk cases.^56,57^

Pelvic radiotherapy (PRT) uses precisely targeted proton beams to destroy tumors while reducing low-dose exposure to surrounding normal tissues via the Bragg peak effect, making it the most conformal external beam technique available. However, studies have shown no clear advantage of PRT over IMRT in terms of quality of life or survival outcomes for PCa, while noting its higher cost and limited availability.^58^ As a result, current guidelines recommend restricting PRT to clinical trials or select cases with specific anatomic indications.

Other emerging RT techniques include adaptive radiotherapy (ART) and BgRT. ART represents a significant paradigm shift, enabling continuous adaptation of the radiation plan based on tumor-specific feedback. This process incorporates anatomical and physiological changes observed through structural imaging such as MRI, functional imaging such as PET, and patient-reported information.^59^ BgRT integrates real-time biological imaging with precise radiation delivery to optimize tumor targeting while sparing healthy tissues, and relies heavily on functional imaging modalities for guidance.^60^ For instance, the RefleXion^®^ X1 system integrates a PET detector with a linear accelerator (LINAC) to enable SCINTIX^®^ therapy, where real-time PET emissions from the tumor itself are used to guide radiation beams for biologically adaptive treatment delivery.^61–63^

### 2.4 | Clinical challenges in PCa radiotherapy

#### 2.4.1 | Toxicity and adverse reactions

The therapeutic benefits of RT are counterbalanced by the risk of acute and chronic toxicities, most commonly affecting the genitourinary and gastrointestinal tracts, as well as contributing to sexual dysfunction.^64–68^ IMRT and IGRT have reduced the incidence of severe late rectal toxicity compared with older 3D-CRT techniques,^69^ particularly when strict rectal dose constraints are applied. Nevertheless, treatment-related toxicity remains a clinical concern. While dose escalation has been shown to improve biochemical control and overall survival (OS) in high-risk PCa patients, these gains often come at the cost of increased toxicity.^70^ Therefore, minimizing unnecessary irradiation of normal tissues is a critical goal. Achieving this requires strategies that can characterize the morphology, biology, and anatomy of cancer spread with greater precision, enabling more accurate treatment planning and safer dose delivery.

#### 2.4.2 | Accuracy in target volume delineation

Accurate target volume delineation is essential in PCa RT to ensure optimal tumor control while minimizing toxicity to adjacent organs at risk. However, consistent and precise contouring of the prostate and potential intraprostatic lesions remains a significant challenge. Due to its limited soft-tissue contrast, CT has difficulty clearly differentiating the prostate from surrounding structures, especially at the apex and base, as well as in identifying extracapsular extension and seminal vesicle involvement. Consequently, CT-based prostate contouring results in volumes that are, on average, 30% larger than the true anatomical volume and encompass only about 84% of the actual gland.^71^ The integration of mpMRI into RT planning has markedly improved visualization of the prostate gland, zonal anatomy, and target definition, while also reducing acute toxicity.^72,73^ Nonetheless, MRI has a false-negative rate of approximately 35% for lesion detection and frequently underestimates tumor volume.^74^ Significant interobserver variability also persists, driven by factors such as vague definitions of clinical target volume (CTV) margins, positional variations of the prostate due to bladder distension and rectal gas, and registration errors between MRI and CT datasets. Delineating target volumes in postoperative setting presents an even greater challenge, as surgically induced anatomical alterations can result in “target invisibility.”^75^ Advanced imaging techniques, especially PSMA PET,^76^ along with emerging AI-based autosegmentation tools,^77^ hold significant promise for improving the accuracy, consistency, and efficiency of target delineation in PCa RT.

#### 2.4.3 | Inadequate individualized treatment

Despite advances in RT techniques for PCa, treatment individualization remains suboptimal. First, dose distribution lacks precise biological tailoring. Although current guidelines recommend risk-stratified treatment for PCa, a “one-size-fits-all” dosing regimen is still applied in some patients, overlooking intertumoral biological heterogeneity. While techniques like IMRT, VMAT, and PRT enable conformal dose shaping, yet there is no consensus on adjusting prescriptions to account for focal tumor aggressiveness, such as dominant intraprostatic lesions (DILs), or to optimally spare dose-limiting organs.^78,79^ Furthermore, ART, which modifies treatment based on anatomical changes during the treatment course, is not yet universally implemented or optimized for biological adaptation.^80^ Growing evidence supports the utility of PET imaging for early response assessment following PCa RT, positioning it as a promising tool to facilitate biologically adaptive ART.

Second, integration of systemic therapy with RT remains insufficiently personalized. While combining RT with systemic therapies, including ADT, chemotherapy, and novel hormonal agents, is standard for higher risk disease, there is a lack of consistent strategies for patient selection, optimal sequencing, and treatment duration.^81–84^ Most clinical decisions are guided by broad risk categories and trial results derived from heterogeneous patient populations.^85^ Genomic classifiers including Decipher, Prolaris, and Oncotype DX have shown potential in refining treatment decisions, including RT timing and the need for adjunct hormone therapy.^86,87^ However, their use in clinical practice remains limited, and these tools are not yet routinely integrated into treatment planning to achieve true personalization.^88^

## 3 | THE ROLE OF PET IN RT PLANNING AND MANAGEMENT OF PCA

### 3.1 | Diagnostic value of PET imaging in biochemical recurrence after radiotherapy

**FIGURE 2:**
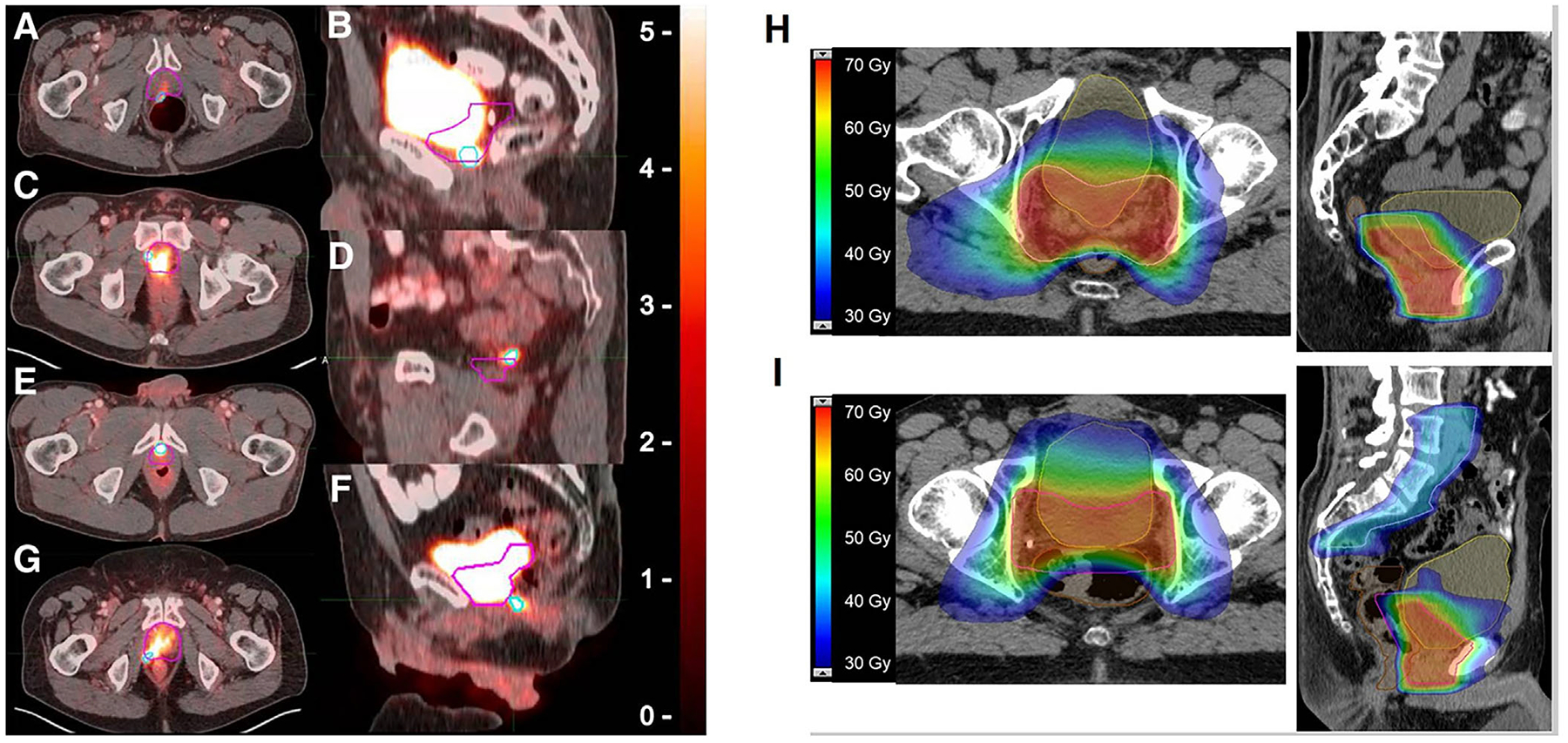
(A–G) Representative cases of [^68^Ga]Ga-prostate-specific membrane antigen (PSMA) positron emission tomography (PET)/computed tomography (CT)-positive prostate-bed recurrences extending beyond the standard Radiation Therapy Oncology Group (RTOG) clinical target volume (CTV). Light blue contours: PSMA-PET-defined lesions. Pink contours: RTOG-based CTV. Panels A–G show the specific directions of lesion extension beyond the CTV: A, Posterior; B, Inferior; C, Lateral; D, Superior; E, Anterior; F, Posteroinferior; G, Posterolateral. Most uncovered recurrences extended posteriorly, posterolaterally, or inferiorly, indicating that current RTOG contouring guidelines may undercover these regions. Reproduced from Ref. 92 with permission from the Society of Nuclear Medicine and Molecular Imaging, copyright 2023. (H, I) Representative salvage radiotherapy (sRT) treatment plans guided by PSMA-PET/CT. Axial (H) and sagittal (I) planning CT images from two prostate cancer patients following radical prostatectomy show color-wash isodose distributions (30–70 Gy) and target delineation. The planning target volume (PTV) is outlined in pink, the rectum in brown, and the bladder in yellow. Patient A: Pathology pT2b pN0 (0/26), ISUP 3, PSA 8.5 ng/mL. Pre-sRT multiparametric MRI (mpMRI) and [^68^Ga]Ga-PSMA PET/CT revealed no local or distant disease. sRT delivered 66.6 Gy to the prostate bed in 1.8 Gy fractions. No recurrence was observed after 44 months of follow-up. Patient B: Pathology pT3b pN1 (3/25), ISUP 3, PSA 13.8 ng/mL. Baseline mpMRI and [^68^Ga]Ga-PSMA PET/CT were negative. sRT delivered 66.6 Gy to the prostate bed and 45 Gy to the elective pelvis (1.8 Gy/fraction), with positive nodes boosted to 54 Gy, plus 6 months of androgen-deprivation therapy. Biochemical recurrence occurred at 31 months, and [^68^Ga]Ga-PSMA PET/CT detected a solitary vertebral bone metastasis. Reproduced from Ref. 93 with permission from the Springer Nature, copyright 2023.

Current sRT contouring guidelines are largely based on expert consensus rather than high-level evidence.^89^ Molecular imaging-guided approaches, particularly with PSMA PET, have been shown to facilitate more appropriate treatment intensification and may also yield cost savings by optimizing resource allocation.^90^ Numerous studies have confirmed the impact of PSMA PET/CT on sRT planning. For example, Solanki et al.^91^ reported that real-time, image-guided, focal dose-escalated high-dose-rate brachytherapy-guided by fused PSMA PET/CT and mpMRI was a feasible and safe salvage strategy for patients with intraprostatic recurrence following prior RT. In another study, Sonni et al.^92^ compared PSMA PET-based contours with those derived from Radiation Therapy Oncology Group (RTOG) guidelines, revealing that standard RTOG definitions often underestimated the extent of recurrence, particularly along posterior and inferior margins. These findings highlight the limitations of conventional contouring protocols and support the integration of PSMA PET into individualized sRT planning and CTV delineation (Figure 2A–G).

Biochemical recurrence following RT is traditionally defined by the Phoenix criteria, characterized by a PSA rise of ≥2.0 ng/mL above nadir. However, multiple large-cohort studies demonstrate that PSMA PET/CT achieves high detection rates even in patients with PSA levels below this threshold^93–95^ (Figure 2H–I). One study found that [^68^Ga]Ga-PSMA-11 PET/CT exhibits high sensitivity for detecting intraprostatic radiorecurrent disease, including in patients previously classified as negative on choline PET/CT, with diagnostic performance comparable to mpMRI.^95,96^ Notably, the combination of PSMA PET/CT with mpMRI significantly enhances both sensitivity and negative predictive value compared with mpMRI alone. A systematic review and meta-analysis by Subiela et al.^97^ concluded that PSMA-ligand PET/CT offers significant clinical value in detecting recurrent PCa, including in patients who do not meet the traditional Phoenix criteria for biochemical failure. The frequent identification of isolated local recurrence at PSA levels ≤2 ng/mL supports timely salvage interventions and warrants reevaluation of the Phoenix threshold in the PSMA PET era.

In a large multinational dataset, Zamboglou et al.^98^ compared PSMA PET-guided sRT cohort (*n* = 462) with a conventional imaging-guided control cohort from the SAKK 09/10 phase 3 trial (*n* = 255). They found that PSMA PET-guided sRT offers short-term improvements in biochemical recurrence-free survival (BRFS), likely due to improved nodal targeting and patient selection, although no significant gain in metastasis-free survival was observed, possibly reflecting earlier detection rather than prevention of metastases. These results underscore the need for further refinement of systemic treatment approaches, such as extended ADT or combination strategies. Similarly, Spohn et al.^99^ evaluated 100 patients with node-positive PCa treated with PSMA PET-guided RT and reported that PSA ≥0.5 ng/mL, M1a disease, or ≥5 PSMA-positive lesions at baseline were associated significantly higher recurrence risk.

**FIGURE 3:**
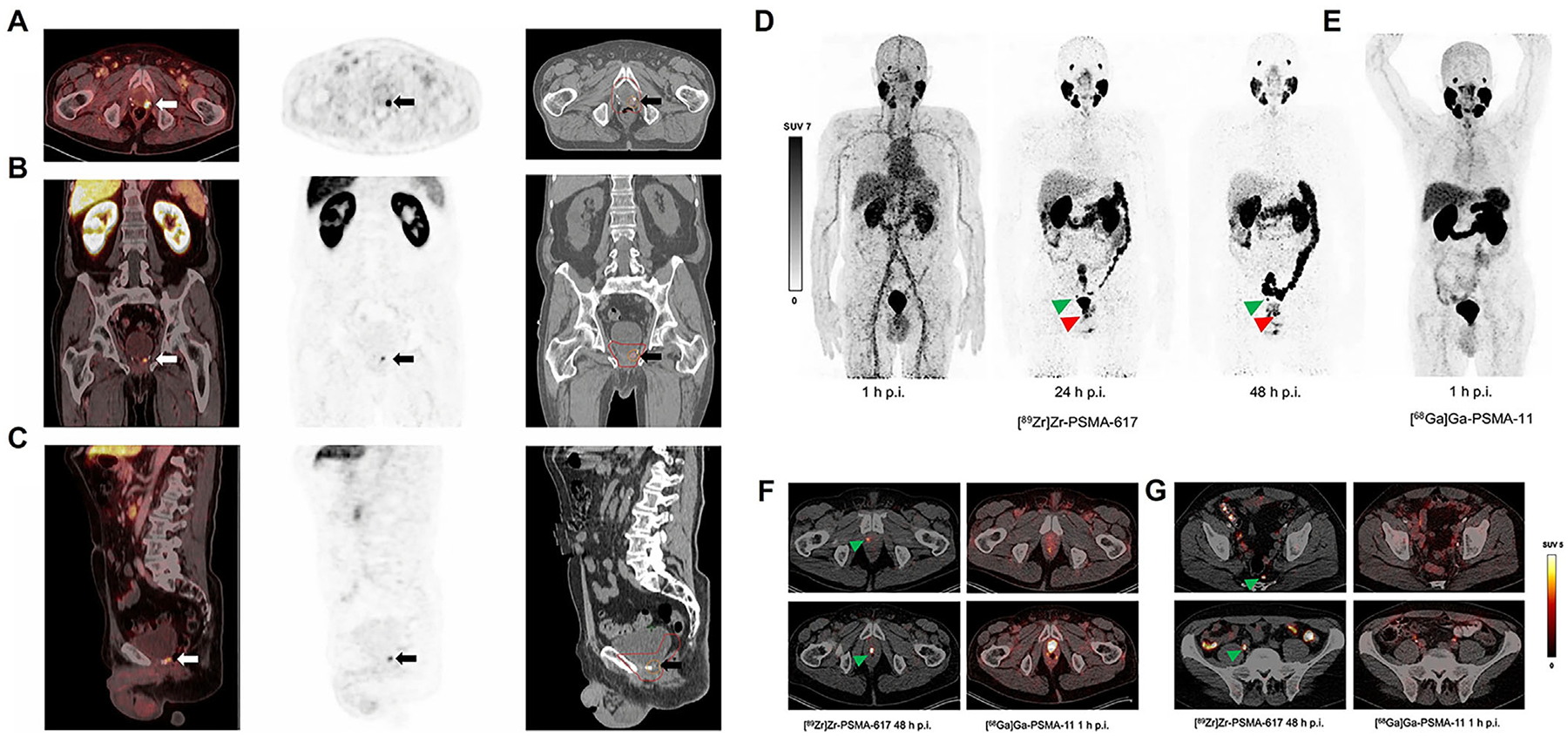
(A–C) [^18^F]-flotufolastat positron emission tomography (PET)/computed tomography (CT)-guided salvage radiotherapy for local prostate cancer recurrence caudal to the bladder. Axial (A), coronal (B), and sagittal (C) PET/CT fusion images show focal uptake (arrows) with corresponding radiotherapy planning contours: red = planning target volume, orange = simultaneous-integrated boost, pink = gross tumor volume. Ref. 93 with permission from the Springer Nature, copyright 2025. (D, E) Maximum-intensity projection PET/CT images of a patient with biochemical recurrence of prostate cancer (PSA 2.5 ng/mL, PSA doubling time >12 months). (D) [^89^Zr]Zr-PSMA-617 PET/CT at 1, 24, and 48 h postinjection shows focal uptake indicating a local recurrence (red arrow) and a pelvic lymph node metastasis (green arrow). (E) [^68^Ga]Ga-PSMA-11 PET/CT at 1 h postinjection fails to reveal these lesions, highlighting the superior detection sensitivity of [^89^Zr]Zr-PSMA-617 for delayed imaging. (F) Transversal PET/CT images of two patients with presumed local prostate cancer recurrence. [^89^Zr]Zr-PSMA-617 PET/CT at 48 h postinjection (left column) clearly identifies focal uptake at the recurrence site (green arrows), whereas [^68^Ga]Ga-PSMA-11 PET/CT at 1 h postinjection (right column) shows no corresponding lesion. (G) Transversal PET/CT images of two patients with suspected pelvic lymph node metastases. [^89^Zr]Zr-PSMA-617 PET/CT at 48 h postinjection (left column) reveals metastatic lymph nodes (green arrows) that are not detected by [^68^Ga]Ga-PSMA-11 PET/CT at 1 h postinjection (right column), demonstrating the enhanced sensitivity of delayed [^89^Zr]Zr-PSMA-617 imaging. Ref. 93 with permission from the Springer Nature, copyright 2023.

Choline PET/CT, utilizing [^11^C]- or [^18^F]-labeled choline tracers, has demonstrated superior sensitivity and specificity over conventional imaging in localizing recurrence. Choline PET retains clinical value in patients with high PSA levels or in regions with limited imaging availability, but PSMA PET/CT offers superior sensitivity and specificity, particularly for detecting low-PSA recurrence and guiding precise treatment decisions.^100^ Detti et al.^101^ reported that [^18^F]-choline PET/CT enabled earlier and more precise treatment decisions in BCR. In a retrospective single-center analysis, [^18^F]-rhPSMA-7 and [^18^F]-flotufolastat PET-guided sRT have shown promising disease control trends in retrospective series,^102^ with a consistent pattern toward improved biochemical failure-free survival (bFS) despite not reaching statistical significance (Figure 3A–C).

Beyond short-lived PET tracers such as ^68^Ga and ^18^F, longer lived isotopes may enhance detection in challenging cases. Rosar et al.^103^ evaluated [^89^Zr]Zr-PSMA-617 PET/CT at 1, 24, and 48 h postinjection and identified suspicious lesions in 78% (18/23) of patients with previously negative [^68^Ga]Ga-PSMA-11 scans. This approach could improve patient selection and enable pretherapeutic dosimetry for PSMA-targeted radioligand therapy, particularly [^177^Lu]Lu-PSMA-617. Prospective multicenter studies with histopathological confirmation are warranted (Figure 3D–G).

Importantly, up to 10% of PCa cases exhibit absent or low PSMA expression, limiting PSMA PET’s utility. Recognizing this heterogeneity, Duan et al.^104^ proposed targeting the gastrin-releasing peptide receptor (GRPR) using [^68^Ga]Ga-RM2 PET/MRI imaging, which markedly enhanced the detection of recurrent PCa in patients with biochemical recurrence and negative conventional imaging. These results position GRPR-targeted PET/MRI as a promising complementary or alternative approach in PSMA-negative disease. Overall, PSMA-PET shows higher detection rates and sensitivity in most clinical settings, but GRPR-targeted PET (RM2/NeoB) can identify additional lesions in patients with PSMA-negative or low-uptake disease, underscoring its complementary value when sued alongside PSMA PET.^105,106^

### 3.2 | PET-guided target delineation and dose optimization strategies

Stereotactic ablative body radiotherapy (SABR) is increasingly employed in PCa management due to its therapeutic efficacy and enhanced patient convenience. The biological characteristics of PCa, particularly its low alpha/beta ratio, render it well suited for hypofractionated treatment regimens. However, conventional treatment planning, which relies heavily on simulation imaging, can prolong the clinical workflow. The integration of PSMA-based molecular imaging into treatment planning, especially for defining biologically relevant target volumes, enables a more refined and individualized approach. This strategy enables precise tumor localization, enhances treatment accuracy, spares surrounding healthy tissue, and may reduce radiation-induced toxicity.^107^ For example, Chatterjee et al.^108^ demonstrated that [^18^F]-choline PET/CT can guide focal dose escalation within a hypofractionated protocol. In high-risk patients with substantial tumor burden, a 20-fraction regimen incorporating PET-directed focal boosting achieved excellent tumor control with acceptable safety.

**FIGURE 4:**
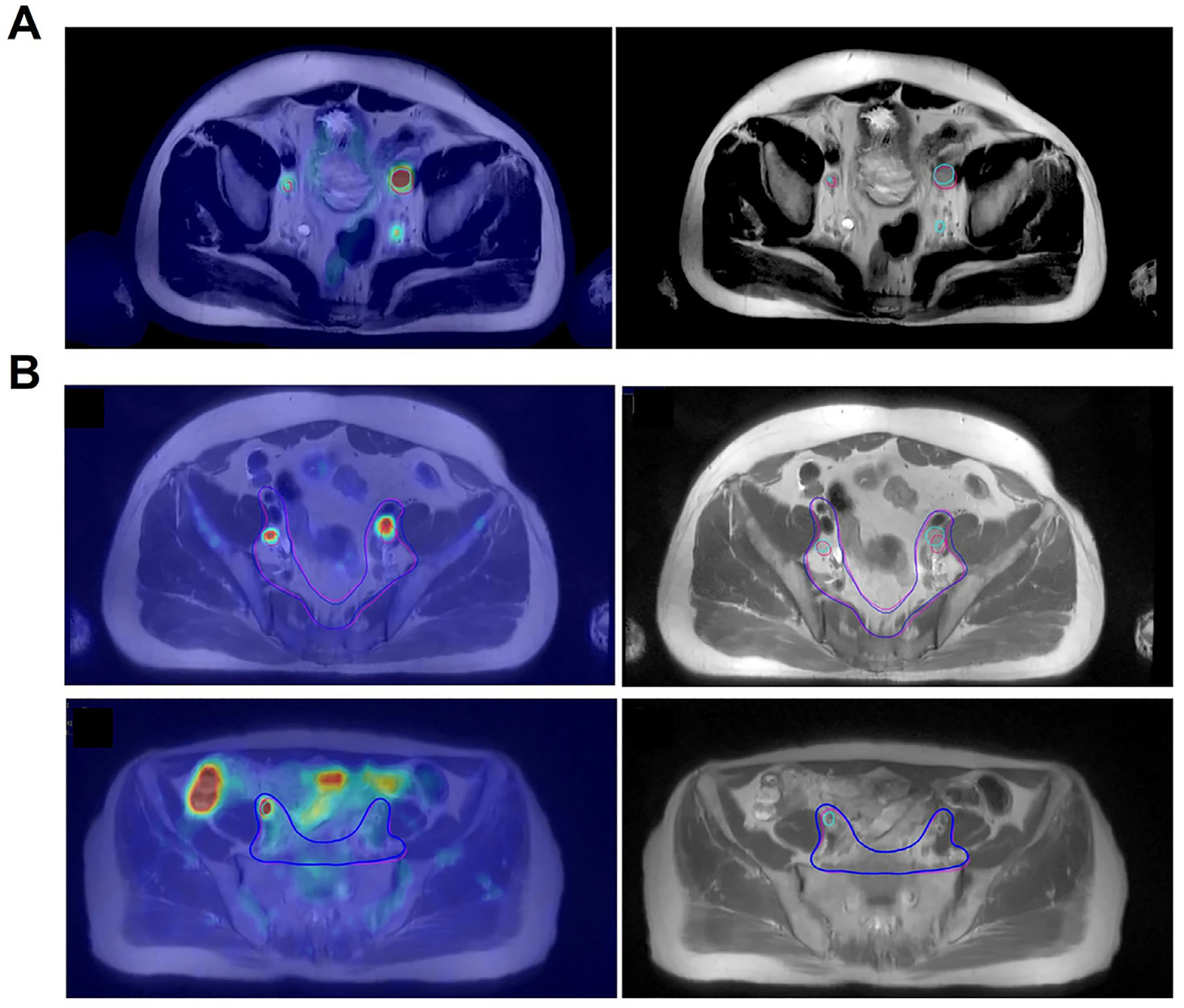
(A) Representative target-volume delineation in a 68-year-old man with high-risk prostate cancer. Prostate-specific membrane antigen (PSMA) positron emission tomography (PET)/magnetic resonance imaging (MRI) identified three pelvic lymph node metastases (green contours), whereas MRI alone detected only two (pink contours), illustrating improved nodal visualization for radiotherapy planning. (B) Examples of whole-pelvis radiotherapy target delineation in high-risk prostate cancer. PSMA PET/MRI (green/blue contours) more accurately reveals nodal metastases and corresponding clinical target volumes than MRI alone (pink contours), highlighting improved precision in lymph node mapping. Ref. 109 with permission from the Springer Nature, copyright 2023.

PSMA PET/CT and PET/MRI have substantially improved the detection of nodal metastases in PCa, leading to significant refinements in RT planning^109–111^ (Figure 4). Building on this, de Leon et al.^112^ explored a simulation-free (sim-free) workflow by integrating diagnostic PSMA PET/CT with MR-LINAC-based adaptive RT in 15 PCa patients. They found minimal dosimetric differences between sim-free, simCT, and first-fraction plans, confirming the feasibility and safety of sim-free MR-guided SABR. Similarly, Ramadan et al.^113^ combined PSMA PET and MRI to localize DILs and involved lymph nodes, delivering SABR with a simultaneous in-field boost (SIB) over five fractions. This approach demonstrated feasibility, safety, favorable biomarker responses, and low rates of acute and late toxicity, with quality of life impacts largely related to hormonal therapy. These findings support the potential of PSMA PET/MRI-guided SABR with focal boosting to optimize disease control while maintaining patient tolerability, warranting further validation in multicenter trials.

In a single-institution study, Murthy et al.^114^ evaluated 170 high-risk PCa patients meeting “STAMPEDE-like” criteria who underwent PSMA PET staging followed by hypofractionated external beam RT with a median equivalent dose (EQD2) of 82 Gy to the prostate, along with long-term ADT for a minimum of 2 years. Outcomes suggested that optimized local therapy with high-dose RT and LT-ADT achieves excellent long-term results, potentially reducing the need for early intensification with agents such as abiraterone. These results highlight the value of incorporating PSMA PET/CT into trial designs for patient stratification and imaging-based endpoints, particularly in oligometastatic and high-risk populations.^115^

Ehret et al.^116^ demonstrated that integrating PSMA PET with mpMRI enables precise target delineation in single-fraction SBRT, providing a safe, effective, and time-efficient salvage strategy for local PCa recurrences, even in patients with extensive prior treatments. Similarly, Menne Guricová et al.,^117^ in a large prospective cohort, found that focal dose escalation to the gross tumor volume (GTV) significantly improved biochemical disease-free survival without increasing toxicity. Spohn et al.^118^ further confirmed the safety and feasibility of PSMA-PET/mpMRI-guided focal dose escalation in both moderately hypofractionated external beam radiotherapy (MHRT) and high-dose-rate brachytherapy (HDR-BT) settings. Grefve et al.^119^ reported that combining PSMA PET with mpMRI improved lesion coverage and reduced interobserver variability in GTV delineation, surpassing either modality alone or conventional CTV margin approaches. These collective findings support the initiation of prospective clinical trials to assess oncological outcomes associated with PSMA-PET/mpMRI-guided focal boost RT. Additionally, Bock et al.^120^ found that [^68^Ga]Ga–PSMA PET/CT altered RT planning in 63% of sRT cases and 9% of definitive radiotherapy (dRT) cases, primarily through target volume expansion and PET-guided boost implementation.

Traditional elective nodal radiotherapy (ENRT) templates, such as the 2009 RTOG guidelines and the 2015 PIVOTAL modifications, were based largely on anatomical landmarks and conventional imaging techniques. PSMA PET/CT, with its superior sensitivity and specificity for detecting lymph node metastases, has exposed significant limitations in these historical templates. In response, the NRG Oncology group released an updated ENRT template in 2021 to refine nodal coverage based on contemporary imaging data.^121–123^ Trapp et al.^124^ compared the RTOG, PIVOTAL, and NRG templates in covering PSMA PET/CT-positive lymph nodes in patients with recurrent PCa after prostatectomy. Although the NRG template significantly reduced the rate of geographic misses compared with earlier versions, it still failed to cover nearly one-third of PSMA-positive nodes, leaving approximately half of patients with inadequate nodal coverage even under the most advanced template. These findings highlight the critical need for individualized RT planning guided by high-resolution molecular imaging such as PSMA PET/CT, rather than fixed anatomical field definitions.

### 3.3 | PET-guided radiotherapy for oligometastases and treatment intensification

Patients with limited metastatic burden are increasingly considered for high-dose, targeted RT, a strategy that has garnered significant interest among radiation oncologists. SBRT-based MDT offers an effective, low-toxicity approach for delaying systemic treatment initiation in oligometastatic PCa. Metabolic imaging-guided SBRT, using PSMA or choline PET, has emerged as a safe and efficacious strategy for achieving durable local control and prolonging systemic therapy-free intervals in selected patients.^125,126^ The largest prospective multicenter trial to date followed 199 patients with oligometastatic PCa for 5 years after SBRT-based MDT.^127^ PSMA PET was integral for staging and lesion identification, with over 75% of patients undergoing PSMA PET imaging prior to treatment. At 5 years, 21.7% of participants remained free from treatment escalation, with comparable outcomes in those with one to three lesions versus four to five lesions. Notably, individuals staged with PSMA PET demonstrated numerically superior outcomes, emphasizing the clinical value of advanced molecular imaging in precision RT. These findings suggest that PSMA PET could play a key role in optimized timing and target delineation for SBRT planning.^128^

Zuur et al.^129^ described the TRACE-II randomized controlled trial, designed to evaluate whether combining PSMA-radioguided surgery (RGS) with short-term ADT improves clinical progression-free survival (CPFS) in patients with oligorecurrent PCa, compared to ADT alone. Completion is expected before December 2025. In a related feasibility study, Hrinivich et al.^130^ assessed the feasibility and dosimetric performance of BgRT using PSMA-targeted PET imaging in patients with oligometastatic PCa. Comparing conventional stereotactic ablative radiation therapy (CSABR), research SABR without PET tracking (RSABR), and PET-guided BgRT, they found that BgRT achieved comparable target coverage (V95% ~95%) but delivered significantly higher maximum tumor doses (Dmax ~150% vs. 128% for CSABR, *p* < .001) while reducing the mean organs at risk doses by 10% (*p* = .02). These findings suggest that PSMA-targeted BgRT is technically feasible and may offer clinical advantages over standard SABR, allow real-time intrafraction tracking, and enable biologically guided dose escalation.

de Bie et al.^131^ investigated the short-term oncological outcomes in patients with metachronous, hormone-sensitive oligometastatic PCa identified via PSMA PET/CT and treated exclusively with metastasis-directed radiotherapy (MDRT). While MDRT effectively delayed disease progression in selected patients, it was unlikely to serve as a standalone treatment for the broader population, highlighting the need for refined patient selection and combined therapeutic approaches. The phase 2 PEACE V–STORM trial provided the first randomized evidence comparing ENRT plus short-term ADT with MDT for PET-detected pelvic nodal oligorecurrence after prior radical treatment, establishing an important benchmark for interpreting subsequent nonrandomized series.^132^ In another study, Trapp et al.^133^ compared hemipelvis radiotherapy (HPRT), which targets only the side harboring PSMA PET/CT-positive lymph node metastases, with whole-pelvis radiotherapy (WPRT) in patients with nodal recurrence following prostatectomy. This first multi-institutional matched-cohort analysis showed that HPRT achieved similar short-term oncologic control to WPRT, despite being a more limited treatment. However, differences in baseline risk factors, such as higher rates of PSA persistence and local recurrence in the WPRT group, suggest that further investigation is needed to guide individualized treatment selection.

**FIGURE 5:**
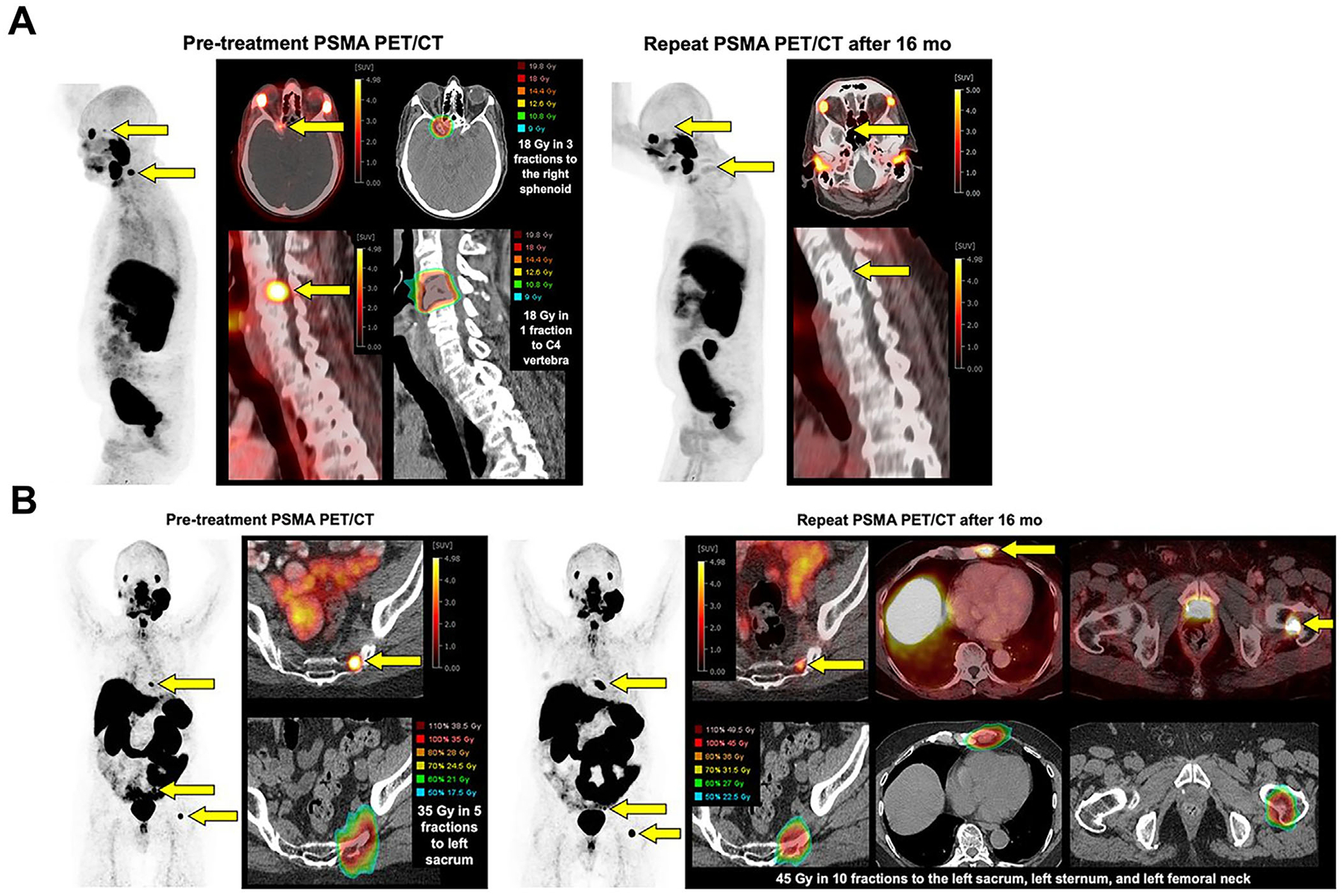
Sequential prostate-specific membrane antigen (PSMA) positron emission tomography (PET)/computed tomography (CT) imaging of metastatic prostate cancer before and after targeted radiotherapy. (A) New metastases in the C4 vertebra and right sphenoid bone (arrows) on baseline imaging, with follow-up showing resolution after metastasis-directed radiotherapy but subsequent multifocal progression. (B) New left sacral metastasis with stable lesions in the left femur and sternum (arrows). Follow-up imaging shows in-field failure in the sacrum and new progression in the left femur and sternum after initial radiotherapy. Reproduced from Ref. 92 with permission from the Society of Nuclear Medicine and Molecular Imaging, copyright 2024.

Metz et al.^134^ compared [^18^F]FCH and [^68^Ga]Ga-PSMA PET/CT for guiding MDRT in hormone-sensitive oligorecurrent PCa patients with PSA levels ≤2 ng/mL. PSMA PET/CT provided clear clinical advantages in lesion detection and RT planning, with extended follow-up of 42.2 months confirming sustained clinical benefit. Nikitas et al.^135^ conducted a retrospective analysis of five prospective trials at UCLA between 2016 and 2023, concluding that PSMA PET/CT-guided MDRT is a safe and efficacious strategy for selected patients with metastatic CRPC. This approach enabled durable disease control and delayed systemic therapy initiation, reinforcing the role of PSMA PET/CT in accurately identifying and targeting oligometastatic lesions while minimizing treatment burden (Figure 5).

### 3.4 | Long-term outcomes and prognostic evaluation of PET-guided radiotherapy

**FIGURE 6:**
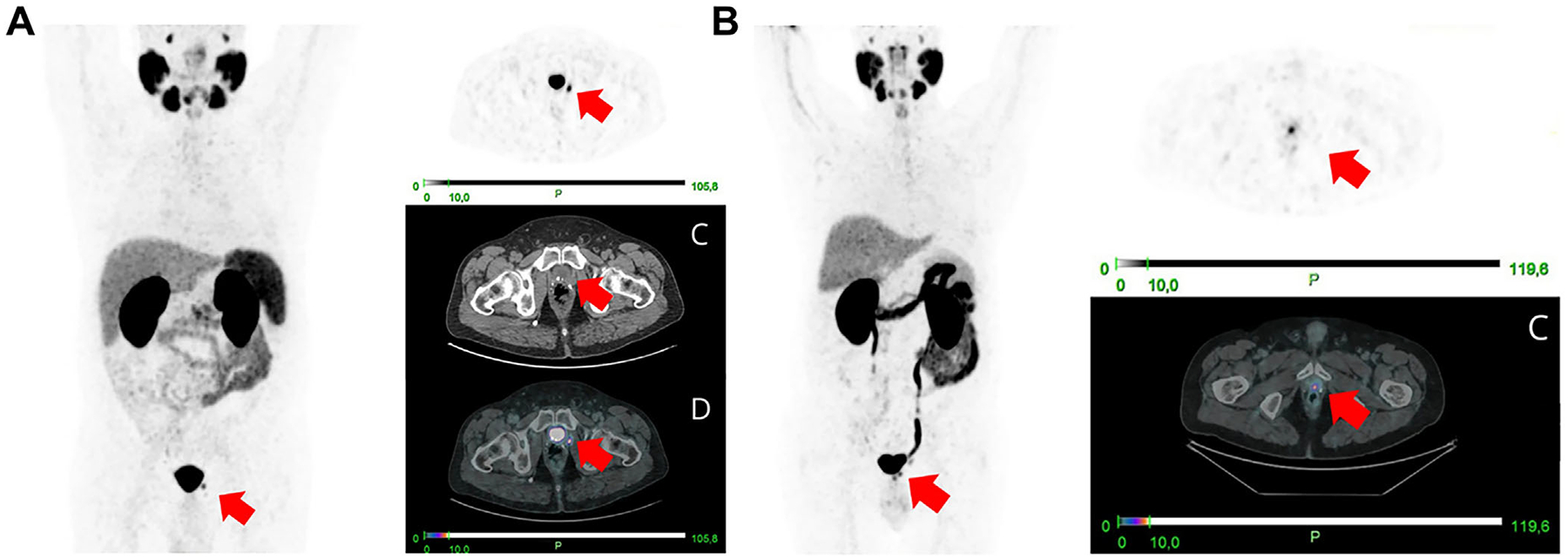
[^68^Ga]Ga-prostate-specific membrane antigen (PSMA) positron emission tomography (PET)/computed tomography (CT) detection of local prostate cancer recurrence after radical prostatectomy. (A) Intense uptake (SUVmax = 11.6) in the prostate bed (red arrow) of a 68-year-old man with biochemical recurrence. (B) Focal uptake (SUVmax = 7.1) in the prostate bed (red arrow) of a 75-year-old man with biochemical recurrence. Reproduced from Ref. 136 with permission from the Springer Nature, copyright 2025.

Approximately 20%–40% of patients experience biochemical recurrence following RP, with sRT increasingly guided by PSMA PET/CT imaging. Di Giorgio et al.^136^ demonstrated that [^68^Ga]Ga-PSMA-11 PET/CT enhances lesion localization, thus refining RT targeting in patients with recurrent or persistent PCa. Their findings indicate that PSMA PET/CT-guided sRT is effective for both biochemical recurrence and persistent PSA (PERS) after RP, producing sustained PSA responses and informing subsequent treatment decisions (Figure 6). In a multicenter retrospective analysis across four high-volume Dutch centers, Meijer et al.^137^ developed and internally validated a nomogram to predict short-term oncological outcomes following sRT in patients with PSMA PET/CT-negative biochemical recurrence after RP. The model accurately estimated the 1-year risk of biochemical progression, with those at predicted risk below 10% showing an observed progression rate of only 4.3%. This suggests that many patients in this category may safely avoid intensified or combined treatment regimens, representing a substantial improvement over earlier predictive tools that lacked contemporary imaging integration.

The European Association of Urology (EAU) biochemical recurrence risk stratification system, based on PSA doubling time and ISUP grade, is used to inform postoperative sRT decisions. While previous studies supported its prognostic value, most did not incorporate PSMA PET staging. Scharl et al.^138^ validated the EAU risk classification in a contemporary cohort of PCa patients staged with PSMA PET and treated with sRT following RP. Their findings confirmed the system’s ability to predict bPFS and metastasis-free survival in a PSMA PET-staged population, although OS and cancer-specific survival differences were not significant, likely due to limited follow-up and low event rates. The authors conclude that the EAU system remains a valuable tool for guiding clinical decision-making in the PSMA PET era, though extended prospective validation is warranted.

Although high PSMA expression is common in aggressive PCa, retrospective studies showed PSMA PET/CT negativity in approximately 4.4%–17% of cases.^139–142^ The proPSMA phase 3 trial conducted by Chen et al.^143^ across 10 Australian centers showed that low PSMA uptake in intermediate- to high-risk PCa is rare (3.3%) and does not necessarily predict poor prognosis. This suggests that PSMA negativity does not automatically equal dedifferentiation or poor prognosis; some tumors may simply have biologic variability in PSMA expression while retaining typical adenocarcinoma behavior. Patients with low PSMA expression can still achieve favorable medium-term outcomes following definitive therapy. The study emphasizes that clinical decisions should not rely solely on PSMA uptake, and modalities such as MRI and biopsy remain essential in guiding treatment for this subgroup.

Notably, multicenter data demonstrate that patients with negative PSMA PET scans can still achieve durable biochemical control after sRT.^144^ Furthermore, Roberts et al.^145^ launched the first prospective DIPPER trial to compare early sRT with active surveillance in patients with low-risk biochemical recurrence and negative PSMA PET/CT findings. The trial aims to assess differences in event-free survival, patient-reported outcomes, and cost-effectiveness between the two management strategies, with results expected in or after 2026.

Lawal et al.^146^ investigated the prognostic value of [^18^F]-fluciclovine PET/CT in predicting failure-free survival (FFS) following sRT, demonstrating that PET imaging not only identifies recurrent lesions but also quantifies disease burden, such as the number of involved lymph nodes, which directly correlates with treatment outcomes. In a separate prospective study, adding [^18^F]-fluciclovine PET/CT to conventional imaging significantly improved FFS in select patient subsets, particularly those with PSA levels below 2 ng/mL, no adverse pathological features, and no prior ADT.^147^ These benefits persisted over the long term without increased treatment-related toxicity, even with more frequent pelvic irradiation. The improved outcomes are attributed to enhanced lesion detection and more precise RT field delineation. Fodor et al.^148^ provided long-term results from a prospective study combining extended nodal radiotherapy (ENRT) with [^11^C]-choline PET/CT-guided simultaneous-integrated boost (SIB) in patients with lymph node-recurrent PCa. The study concluded that ENRT with PET-guided SIB offers durable disease control, particularly in hormone-sensitive PCa. Median clinical recurrence-free survival (CRFS) was 67 months, and OS extended to 110 months. Although outcomes were less favorable in the CRPC subgroup, the regimen still provided extended disease control compared to historical benchmarks, indicating the potential benefit of aggressive locoregional therapy even in extensive nodal or advanced disease states.

Despite the increasing use of PSMA PET/CT in RT planning, the post-treatment kinetics of PSMA uptake remains poorly understood. Early post-RT uptake may reflect inflammatory responses or tissue remodeling rather than viable tumor. Hotta et al.^149^ demonstrated that PSMA PET signal intensity declines progressively following RT, with the optimal window for assessment being approximately 8–12 months post-treatment. Imaging performed within 3 months is susceptible to misinterpretation due to pseudo-progression. Lesion location and baseline tumor burden further influence tracer kinetics, underscoring the need for careful timing and anatomical context when interpreting post-treatment scans. These findings support a cautious approach to early post-RT imaging and suggest that persistent uptake does not always signify active disease.

In a large multicenter retrospective study, Bauckneht et al.^150^ compared outcomes from patients undergoing [^68^Ga]Ga-PSMA-11 PET/CT versus choline PET/CT. The study found that PSMA PET/CT was associated with longer progression-free survival, delayed initiation of systemic therapy, and improved OS. These results emphasize that PSMA and choline PET/CT are not interchangeable in this clinical setting of PET-detected oligorecurrent prostate cancer after radical treatment, where patients are being evaluated for sRT or MDT, and that imaging modality choice can directly impact long-term clinical outcomes beyond diagnostic accuracy.

**FIGURE 7:**
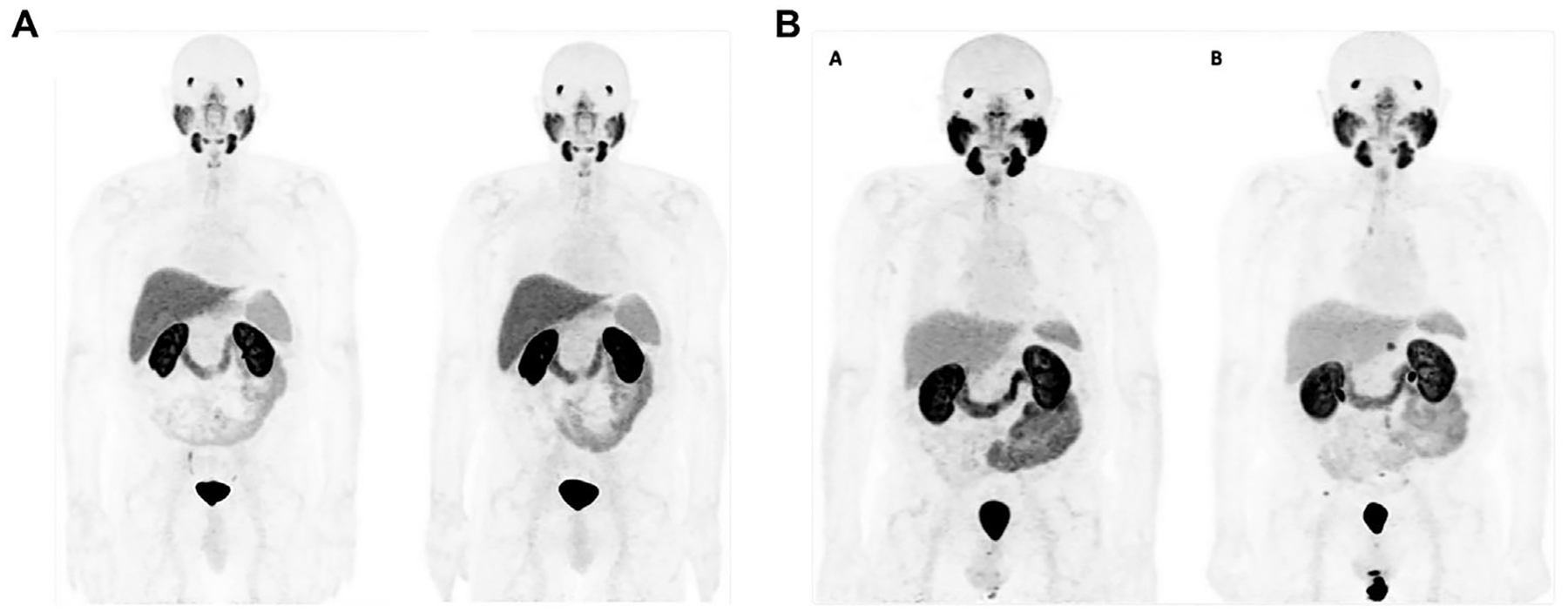
Sequential [^18^F]DCFPyL prostate-specific membrane antigen (PSMA) positron emission tomography (PET)/computed tomography (CT) scans demonstrating different biochemical recurrence patterns after radical prostatectomy. (A) Patient with stable PSA and no detectable disease on both baseline and 25-month follow-up imaging. (B) Patient with initial negative baseline imaging but, after 28 months, development of multiple PSMA-avid lymph node and osseous metastases, indicating disease progression. Reproduced from Ref. 152 with permission from the Springer Nature, under CC BY 4.0 license, copyright 2025.

Harsini et al.^151^ purposefully excluded systemic therapy to isolate the therapeutic impact of PSMA PET/CT-guided MDT in patients with biochemical recurrence. Among patients with five or fewer PSMA-positive lesions and no ADT, localized treatment based on PSMA PET/CT findings yielded robust PSA responses and encouraging bPFS. In follow-up work, the same group found that even patients with negative PSMA PET/CT scans benefited from early sRT post-RP, achieving improved freedom from clinical progression^152^ (Figure 7).

Henríquez et al.^153^ identified maximum standardized uptake value (SUVmax) on PSMA PET/CT as a prognostic biomarker in metastatic hormone-sensitive PCa. Their findings showed that SUVmax predicts treatment response to androgen receptor signaling inhibitors (ARSIs) combined with ADT. Similarly, Ades et al.^154^ assessed the prognostic significance of intraprostatic SUVmax in patients with nonmetastatic PCa undergoing curative-intent therapy via RP or RT. The study found that SUVmax >5.6 was independently associated with worse bFS (Hazard Ratio (HR) = 4.4, *p* = .005). This association persisted after adjusting for age and ISUP grade. Their use of regression tree analysis to define actionable SUVmax thresholds offers a practical tool for risk stratification and treatment planning, though validation in larger, long-term cohorts is warranted. Recent data^155,156^ also highlight the prognostic significance of PSMA PET/CT in localized prostate cancer, further supporting the role of SUVmax as a biomarker across disease stages.

Onal et al.^157^ investigated early metabolic response using [^68^Ga]Ga-PSMA PET/CT in a uniformly defined cohort of intermediate-risk PCa patients following RT, observing significant early reductions in PSMA expression and serum PSA levels, especially when RT was combined with ADT. However, early [^68^Ga]Ga-PSMA PET/CT imaging alone was insufficient for treatment response evaluation in patients receiving RT only. Its most promising role may lie in identifying those reaching PSA nadir post-RT combined with ADT, aiding early response stratification. In related work, metabolic response after neoadjuvant ADT (nADT) on [^68^Ga]Ga-PSMA-11 PET/CT was shown to be a valuable prognostic indicator in high-risk PCa patients undergoing RT.^158^

In a separate multicenter retrospective study, Onal et al.^159^ reported that PSMA PET/CT-guided staging in clinically node-positive PCa treated with definitive RT and ADT was linked to improved PCa-specific survival. Longer ADT duration and favorable PSA responses were key predictors of better outcomes. These findings support broader integration of PSMA PET/CT into the staging and planning of node-positive PCa and highlight future research directions, including evaluation of metabolic tumor volume as a prognostic marker and exploration of novel systemic therapies in patients with high-risk features or suboptimal PSA responses.

## 4 | PERSPECTIVES AND FUTURE DIRECTIONS

PSMA PET has revolutionized PCa imaging and clinical management by enabling earlier detection of recurrence, more accurate staging, and improved treatment stratification.^160^ It also plays a critical role in guiding and monitoring PSMA-targeted radioligand therapy. However, the successful integration of PSMA PET/CT into clinical workflows requires close interdisciplinary coordination, along with standardized imaging protocols and interpretation criteria. The PROMISE criteria were developed to address these needs by standardizing reporting, enhancing clinical decision-making, and providing objective assessments of oncologic response.^161^

As RT becomes increasingly precise and conformal, the establishment of clear guidelines and uniform protocols is vital to reduce interobserver variability and ensure consistent target volume delineation.^10^ One inherent limitation of PET is its lack of anatomical context. This limitation has been effectively mitigated by hybrid imaging modalities such as PET/CT and PET/MRI, which enable accurate localization of molecular signals within an anatomical framework.^162^

PET molecular imaging is playing an increasingly pivotal role in the PCa RT planning, transforming treatment paradigms through enhanced patient selection, refined dose optimization, and improved disease control. Moreover, it offers a unique opportunity to monitor the temporal evolution of tumors throughout the treatment course. In recent years, a variety of novel molecular biomarkers have been developed and validated across diverse PCa contexts. Concurrently, advances in tracer development, including improved pharmacokinetics and shortened imaging protocols, are enhancing clinical applicability. Nonetheless, robust evidence from multicenter, prospective trials is essential to support widespread adoption of these innovations.

One particularly promising advancement is the integration of PET imaging with LINACs to enable BgRT, a modality that fundamentally differs from conventional IGRT by incorporating real-time biological information into treatment delivery.^9,61–63^ This technique employs dynamic tracking of tumors via partial real-time PET images, with radiotracer emissions serving as biological fiducials. This allows continuous adaptation of radiation delivery without additional positional margins, thereby enhancing precision and minimizing exposure to surrounding healthy tissues.^163^

One notable implementation of this technology is the RefleXion^®^ X1 system (RefleXion Medical, Inc.), a PET-LINAC platform that combines CT and PET imaging with a 6 MV LINAC in a ring gantry configuration. It enables SCINTIX^®^ therapy, in which PET-detected tumor emissions guide radiation beams in real time. By using the tumor itself as a dynamic target, SCINTIX reduces the need for large CTV margins and advances the concept of PET-guided ART.^61–63^ In 2023, SCINTIX therapy received FDA clearance for the treatment of lung and bone tumors, both primary and metastatic, using [^18^F]-FDG. Incorporating PSMA-targeted radiotracers into SCINTIX-guided protocols could provide a new therapeutic avenue for PCa.

Despite its promise, PET-LINAC adoption faces challenges, including motion management, PET signal stability, and the lack of standardized protocols for defining biological tracking zones. At present, PET-LINAC is not widely used as an online guidance tool in routine RT procedures.^163^ High-quality translational research is urgently needed to validate its accuracy and reliability, refine target delineation, and enhance the precision of dose delivery. Furthermore, rigorous studies should also determine optimal strategies for incorporating PET-LINAC into RT workflows. Given the substantial cost of BgRT, strong clinical evidence will be essential to justify its incorporation into standard care and ensure cost-effectiveness.

Emerging radionuclide therapies and advanced image-guided RT offer synergistic strategies for PCa management. ^177^Lu- and ^225^Ac-labeled PSMA radioligand therapies provide systemic, targeted irradiation to PSMA-expressing lesions and have shown meaningful therapeutic benefits in metastatic castration-resistant disease. By contrast, PSMA PET-guided external-beam RT leverages the exceptional sensitivity of PSMA imaging to refine target delineation, enable focal dose escalation, and individualize treatment volumes for localized or oligometastatic tumors, thereby enhancing tumor control while reducing normal-tissue exposure. Rather than being competing modalities, radioligand therapy and PSMA-guided external-beam RT can be used in a complementary manner: radioligand therapy for disseminated disease and PET-directed RT for locoregional control or oligoprogression. This highlights the need for prospective trials to define optimal patient selection and sequencing strategies for combined treatment paradigms.

As clinical evidence and access to advanced imaging grow, PET-guided RT is likely to become a standard component of radiation oncology, offering the potential for improved oncologic outcomes and enhanced patient quality of life. Key research priorities include improving registration accuracy between PET molecular images and anatomical imaging before and after treatment, developing standardized clinical protocols for novel PET tracers in RT planning, streamlining BgRT workflows, and enhancing clinical scalability. Future investigations should also evaluate cost-effectiveness, determine optimal timing for PET integration within treatment workflows, and establish robust, reproducible imaging protocols to support broad clinical adoption.

## 5 | CONCLUSION

The integration of diagnostic imaging and RT is central to the contemporary management of PCa. Recognizing the intrinsic interdependence between these disciplines is essential to understanding both the strengths and limitations of current radiotherapeutic strategies. As highlighted in this review, molecular imaging techniques, particularly PSMA PET, have transformed PCa care by profoundly influencing RT planning and clinical decision-making. Incorporating PET imaging into clinical workflows enhances patient stratification, improves target volume delineation, and informs MDTs. These advances hold promising for improving patient outcomes while minimizing treatment-related toxicity.

PET-based molecular imaging in PCa is evolving rapidly, with emerging technologies poised to further reshape clinical management and RT planning. Among these, BgRT offers a novel opportunity to leverage PET tracers as real-time biological probes for precision treatment. Incorporating PET imaging into BgRT workflows facilitates more accurate definition of treatment volumes, identification of radioresistant tumor regions, and personalization of therapy through refined target delineation, optimized planning, and biologically guided dose escalation. Systematic incorporation of these strategies into future prospective, randomized clinical trials will be critical to establishing their clinical value. The results of ongoing studies in this area are eagerly anticipated.

## Figures and Tables

**TABLE 1:** Summary of studies on PET-guided radiotherapy in prostate cancer.

Years	Research methodology	Number of patients enrolled	PET imaging methodology	Outcomes	Limitations	Refs.
2023	A single-center retrospective analysis	226	[^68^Ga]Ga-PSMA PET/CT	The study concluded that existing RTOG guidelines frequently underrepresent the true extent of recurrence detectable by [^68^Ga]Ga-PSMA PET, particularly at posterior and inferior borders.	Lesion delineation on PET is subject to partial volume and spillover effects due to limited spatial resolution. And the absence of follow-up outcome data (e.g., local control or survival) prevents conclusions on clinical benefit.	Sonni et al.^92^
2023	A single-center retrospective analysis	273	[^68^Ga]Ga-PSMA-11 PET/CT	PSMA-PET/CT-guided sRT is highly effective even at very low PSA levels, with promising BRFS rates and minimal in-field recurrence.	Limitations include its retrospective nature, lack of standardized imaging and treatment protocols across centers, relatively short follow-up duration, and small sample size due to the rare use of PSMA PET/CT at such low PSA levels.	Solomonidou et al.^93^
2025	A multicenter retrospective analysis	568	[^68^Ga]Ga-PSMA-11, [^18^F]-DCFPyL, [^18^F]-PSMA-JK7, [^18^F]-PSMA-1007 PET/CT	PSMA PET/CT is effective in detecting PCa recurrence in patients before they meet the Phoenix criteria. Early detection was associated with a higher chance of salvage treatment, delayed systemic therapy, slower disease progression, and improved survival.	The retrospective design inherently carries risks of selection bias and unstandardized clinical decision-making regarding ADT initiation. The absence of cause-specific mortality data limits conclusions about PCa-specific survival.	Altena et al.^94^
2023	A single-center retrospective analysis	159	[^68^Ga]Ga-PSMA-11 PET/CT	[^68^Ga]Ga-PSMA-11 PET/CT effectively detects recurrent prostate cancer even in patients previously deemed negative by choline PET/CT.	The retrospective design limited access to complete baseline and longitudinal data, and the study population was biased toward high-risk patients, reducing generalizability.	Pinot et al.^95^
2024	A single-center retrospective analysis	35	[^68^Ga]Ga-PSMA-11 PET/CT	The main findings showed that PET/CT had a sensitivity of 0.89 and an NPV of 0.79 for detecting any cancer, compared with MRI’s sensitivity of 0.72 and NPV of 0.59, though these differences were not statistically significant individually. When both modalities were used together, sensitivity significantly increased to 0.98 (*p* = .003) and NPV to 0.93 (*p* < .001), surpassing MRI alone.	The study’s limitations include its small sample size, single-center nature, and retrospective design, which may limit generalizability.	Light et al.^96^
2024	Retrospective multicenter cohort and prospective randomized controlled trial	717	PSMA PET/CT	PSMA PET-guided sRT offers short-term improvement in BRFS compared to conventional imaging-guided sRT, likely due to better nodal targeting and patient selection. However, it does not improve MFS, possibly due to early metastasis detection rather than prevention.	Retrospective bias, follow-up heterogeneity, nonrandomized PSMA PET cohort, and shorter median follow-up.	Zamboglou et al.^98^
2022	A single-center retrospective analysis	100	[^68^Ga]Ga-PSMA-11, [^18^F]-PSMA-1007 PET/CT	Patients with node-positive prostate cancer who present with high PSA levels (≥0.5 ng/mL), M1a disease, or ≥5 PSMA-positive lesions at the time of imaging are at significantly increased risk for biochemical recurrence following PSMA PET-guided RT.	A relatively small sample size, short follow-up duration, lack of central review of PET imaging, and usage of two different PSMA PET tracers, potentially causing variations in results.	Spohn et al.^99^
2023	A retrospective multicenter analysis	410	[^18^F]-choline PET/CT	The conclusions affirm that [^18^F]-choline PET/CT offers superior accuracy over conventional imaging in detecting BR, enabling earlier and more targeted intervention. PET findings—especially the number and location of lesions—are pivotal in determining the optimal management strategy.	The limitations include the retrospective design, possible selection bias, and lack of comparison with PSMA PET/CT in the same cohort.	Detti et al.^101^
2025	A single-center retrospective analysis	110	[^18^F]-rhPSMA-7, [^18^F]-flotufolastat PET/CT	The results show that PET-guided sRT consistently achieved numerically superior bFS rates at all measured timepoints (12, 24, 36, and 48 months).	Retrospective design, small cohort size, shorter follow-up for PET-guided cases, and lack of long-term imaging-based outcomes.	Vogel et al.^102^
2023	A single-center retrospective analysis	23	[^68^Ga]Ga-PSMA-11 PET/CT, [^89^Zr]Zr-PSMA-617 PET/CT	[^89^Zr] Zr-PSMA-617 PET/CT provides a valuable diagnostic option for detecting prostate cancer lesions in biochemical recurrence patients with previously negative conventional scans, even at low PSA levels. Imaging at 48 h postinjection yielded the highest lesion-to-background contrast and revealed additional lesions not seen at earlier time points. These findings support the use of [^89^Zr]Zr-PSMA-617 PET/CT in specific clinical scenarios where conventional tracers fail.	The study’s limitations include its retrospective, single-center nature, and relatively small sample size (*n* = 23), which may limit generalizability.	Rosar et al.^103^
2024	A prospective, single-center, phase 2/3 clinical imaging trial (NCT05555017)	100	[^68^Ga]Ga-RM2 PET/MRI	Sensitivity (85.2% vs. 49.4%), negative predictive value (61.3% vs. 30.5%), and accuracy (88.0% vs. 58.0%) were all significantly better for [^68^Ga]Ga-RM2 PET/MRI, while specificity and positive predictive value were comparable. Subgroup analyses revealed that [^68^Ga]Ga RM2 PET/MRI outperformed MRI particularly in patients with PSA between 0.5–1.0 ng/mL and ≥5.0 ng/mL.	Not all lesions were validated histologically, potentially inflating specificity. Patient heterogeneity regarding PSA levels, tumor burden, and Gleason scores may affect generalizability.	Duan et al.^104^
2023	A single-center retrospective analysis	64	[^18^F]fluoro-JK-PSMA, [^99m^Tc]Tc-mas3-y-nal-k(Sub-KuE) PET/CT	PSMA-based imaging, particularly when used to define biological target volumes for SBRT, significantly alters and refines treatment planning. It allows for more precise targeting, potentially sparing healthy tissue and reducing toxicity.	Patient selection may have been subject to bias, and follow-up PSMA imaging was not available for all participants.	Varga et al.^107^
2024	A single-center prospective study	62	[^18^F]-choline PET/CT	The study concludes that hypofractionated radiotherapy with integrated focal boosts is both feasible and effective, achieving strong tumor control and low GI toxicity. Although urinary toxicity was more common, symptoms generally improved over time and remained manageable.	Limitations include the nonrandomized, single-arm design and modest sample size, limiting direct comparison with standard treatment.	Chatterjee et al.^108^
2023	A single-center retrospective analysis	49	[^18^F]F-PSMA-1007, [^68^Ga]Ga-PSMA-11 PET/MRI	The conclusions drawn indicate that although CTV delineation using PET/MRI does not significantly differ from MRI, PET/MRI provides superior accuracy in GTVn delineation and in identifying true metastatic lymph nodes, leading to more individualized and precise radiotherapy plans.	It was retrospective in design, potentially introducing selection bias. Node-to-node imaging-pathology correlation was not feasible.	Liu et al.^109^
2024	A single-center retrospective analysis	95	[^68^Ga]Ga-PSMA and [^18^F]PSMA-1007 PET/CT	PSMA PET/CT identified additional positive lymph nodes in 48% of patients. This led to a change in nodal staging from N0 to N1 in 29% of patients. Among the 28 patients with PET-only nodal positivity, 18% had field modifications and 83% received dose boosts.	Limitations include its retrospective and single-center nature, which may introduce selection bias and limit generalizability. The lack of long-term oncological outcome data due to limited follow-up duration also restricts conclusions about survival or recurrence.	Furman et al.^110^
2024	A single-center prospective observational study	15	PSMA PET/CT	Sim-free MRgRT is clinically feasible and dosimetrically accurate for prostate SABR, even in a definitive treatment setting.	Small sample size, prolonged initial planning time, lack of full automation, and no standardized bowel/bladder prep in diagnostic scans.	de Leon et al.^112^
2025	A multicenter prospective study	50	[^18^F]-PSMA-1007 PET/MRI	PSMA PET/MRI-guided SABR with focal SIB is feasible and safe, with low acute and late toxicity and favorable biomarker responses.	Dose escalation was constrained by organ-at-risk proximity, and the median delivered dose was below the target 50 Gy. The small sample size and relatively short follow-up (median 12.5 months) limit conclusions on long-term outcomes.	Ramadan et al.^113^
2025	A single-center retrospective analysis	170	PSMA PET/CT	The results suggest that intensified local treatment (high-dose RT and LT-ADT) without additional systemic intensification can achieve comparable outcomes to the intensified systemic treatment in selected high-risk prostate cancer patients staged by PSMA-PET/CT.	Survival outcomes were defined using PSMA-based imaging, which differs from the conventional imaging-based endpoints used in the original STAMPEDE trial, potentially influencing survival interpretation.	Murthy et al.^114^
2023	A multicenter prospective study	64	PSMA PET/CT or MRI	Single-fraction PSMA PET/mpMRI-guided SBRT is a safe, effective, and time-efficient salvage treatment option for local PCa recurrences, even in patients with significant prior treatment history.	Limitations including its retrospective nature, single-center design, relatively short follow-up for assessing long-term outcomes or late toxicity, and a small, heterogeneous patient population.	Ehret et al.^116^
2024	A multicenter prospective study	535	PSMA PET/CT	Nearly all IPRs occurred within the original GTV and in cases where the delivered dose was insufficient. These results reinforce the rationale for aggressive focal boosting and suggest that reducing the CTV dose could be explored as a way to mitigate toxicity without compromising local tumor control.	The timing of imaging was based on biochemical failure, not on a fixed schedule, introducing uncertainty in disease progression chronology. and interobserver variability in image registration and contouring, especially of PSMA PET-defined lesions, could influence localization accuracy.	Menne Guricová et al.^117^
2025	A multicenter prospective study	55	[^68^Ga]Ga-PSMA, [^18^F]F-PSMA-1007 [^68^Ga]Ga-PSMA-11 PET/MRI	The conclusions affirm that focal dose escalation using PSMA-PET and mpMRI is feasible and safe in both MHRT and HDR-BT settings. HDR-BT + EBRT demonstrated lower GI toxicity and better bowel QoL outcomes.	The limitations include the small sample size (*n* = 55), reducing statistical power, and the nonrandomized design, which may introduce selection bias.	Spohn et al.^118^
2024	A single-center prospective study	15	[^68^Ga]Ga-PSMA PET/CT	Combining imaging modalities, particularly PSMA-PET with mpMRI, enhances both the accuracy (lesion coverage) and consistency (lower interobserver variability) of GTV delineations compared to single modalities or the use of CTV margins.	Key limitations include the small sample size (15 patients) and the fact that histopathological delineations were performed by a single pathologist, potentially introducing observer bias.	Grefve et al.^119^
2024	A single-center prospective study	38	[^68^Ga]Ga-PSMA PET/CT	The study concluded that [^68^Ga]Ga-PSMA PET/CT influenced radiation planning in nearly 63% of sRT cases and 9% of dRT cases, mainly by expanding target volumes and adding PET-guided boosts.	Limitations include the small sample size (*n* = 38) and short median follow-up (12 months), which limits conclusions regarding long-term outcomes such as disease-free survival or overall survival.	Bock et al.^120^
2023	A single-center retrospective imaging study	105	[^68^Ga]Ga-PSMA-11 or [^18^F]-PSMA-1007 PET/CT	The NRG template provided the best LN coverage, missing only 31.7% of LNs per patient on average (mean 1.01 LNs), compared to 40.1% with RTOG (mean 1.28 LNs) and 37.3% with PIVOTAL (mean 1.19 LNs). These differences were statistically significant (*p* < .001 for RTOG vs. NRG; *p* = .003 for PIVOTAL vs. NRG).	Patient selection bias exists, with a wide range of PSA levels and inclusion of patients with bone metastases.	Trapp et al.^124^
2023	A single-center retrospective analysis	103	[^68^Ga]Ga-PSMA PET/CT	PSMA PET-guided SBRT is a safe and effective strategy for selected patients with oligometastatic PCa, achieving excellent local control and a substantial delay in the need for ADT. Although only a minority remained completely BF-free at 5 years, over half of the cohort avoided ADT for relapse, indicating meaningful clinical benefit.	Retrospective, nonrandomized design. Potential clinician bias in selecting patients for SBRT versus other therapies.	Mohan et al.^125^
2024	A multicenter prospective study	199	Choline and PSMA PET/CT	SBRT-based MDT is an effective, low-toxicity approach for delaying systemic therapy in oligometastatic PCa. Despite modest absolute TE-FS rates, the ability to postpone ADT for a significant number of patients—particularly those who are hormone-naive—offers a valuable clinical alternative.	There was no comparator arm (e.g., ADT-only group), limiting conclusions about relative efficacy.	See et al.^127^
2020	A single-center, retrospective simulation study	15	[^18^F]-DCFPyL PET/CT	The BgRT plans provided comparable target coverage and conformity to the conventional clinical SABR plans. Specifically, BgRT significantly increased the maximum planning target volume (PTV) dose (by approximately 18%, *p* < .001) and slightly decreased the mean dose to nearby organs at risk (by about 10%, *p* = .02) compared to CSABR.	Small sample size (15 patients), and absence of real-time intrafraction motion tracking validation, as the study did not include actual delivery of radiation but rather simulated treatments.	Hrinivich et al.^130^
2025	A single-center retrospective analysis	80	PSMA PET/CT	MDRT is a safe and effective local therapy for PSMA-detected oligometastatic PCa, achieving excellent local control. However, its ability to prevent systemic progression is limited, as the majority of patients developed new lesions over time.	The study’s shortcomings include its retrospective nature, lack of a control group, and heterogeneity in patient characteristics and imaging protocols.	de Bie et al.^131^
2024	A multicenter retrospective analysis	102	PSMA PET/CT	The conclusions suggest that HPRT, despite being less extensive, achieved similar short-term oncologic outcomes to WPRT. However, WPRT patients might have had higher baseline risk due to more frequent PSA persistence and local recurrences.	The limitations stem from the retrospective nature, lack of standardized treatment protocols, and missing data on factors like number and laterality of lymph node metastases, toxicity profiles, and ADT duration.	Trapp et al.^133^
2023	A single-center retrospective analysis	123	[^18^F]F-choline (FCH) and [^68^Ga]Ga-PSMA PET/CT	The authors concluded that PSMA PET/CT provides clinical advantages over FCH PET/CT for guiding RT in oligorecurrent hormone-sensitive PCa, especially in patients with low PSA levels. It allows better detection and characterization of disease, enabling more targeted and potentially curative RT.	Treatment heterogeneity, lack of randomization, and potential selection bias (e.g., more naive patients in the PSMA group) weaken causal inferences.	Metz et al.^134^
2024	A multicenter prospective study	24	[^68^Ga]Ga-PSMA-11 PET/CT	PSMA PET/CT-guided MDRT is a safe and effective strategy for selected mCRPC patients, offering durable control while postponing systemic treatment escalation.	It is retrospective, includes a small and heterogeneous patient cohort, and involves variable systemic therapy regimens and radiation techniques.	Nikitas et al.^135^
2025	A single-center retrospective analysis	100	[^68^Ga]Ga-PSMA-11 PET/CT	[^68^Ga]Ga-PSMA PET/CT-guided sRT is effective for both biochemical recurrence and PERS after RP, yielding durable PSA responses and helping inform further therapeutic decisions. PSA level prior to sRT is a key prognostic factor, and early intervention in PSA persistence cases with nodal involvement may improve outcomes.	The main limitations include its retrospective design, lack of a control group using conventional imaging-guided sRT, and single-center nature, which may limit generalizability.	Di Giorgio et al.^136^
2024	A retrospective multicenter analysis	302	[^68^Ga]Ga-PSMA-11 PET/CT	The study concludes that their novel nomogram effectively predicts 1-year biochemical progression after sRT in patients with PSMA PET/CT-negative biochemical recurrence post-RP. This is the first tool of its kind to integrate PSMA PET/CT imaging findings, offering clinicians a more accurate method for patient stratification.	The use of various PSMA tracers, imaging protocols, and radiation doses across institutions introduces heterogeneity. And, the model has not been externally validated.	Meijer et al.^137^
2024	A retrospective multicenter analysis	526	[^68^Ga]Ga-PSMA-11or [^18^F]-PSMA-1007 PET/CT	EAU risk stratification is a reliable predictor of outcomes in PSMA-PET-staged patients receiving sRT. Multivariate Cox regression confirmed that EAU risk group independently predicted both BPFS (HR 2.022, *p* = .003) and MFS (HR 2.986, *p* = .013), along with pT stage and resection status.	The follow-up period was relatively short (median 31 months), limiting the ability to assess long-term outcomes like OS and cancer-specific survival.	Scharl et al.^138^
2025	A multicenter prospective study	302	[^68^Ga]Ga-PSMA-11 PET/CT	The study found that only 3.3% (10 out of 302) of the cohort had low intraprostatic uptake (SUVmax ≤ 4), a figure notably lower than in retrospective reports. low PSMA uptake in intermediate- to high-risk prostate cancer is uncommon and not necessarily associated with poor outcomes.	The small sample size of patients with low uptake (*n* = 10) restricts generalizability.	Chen et al.^143^
2024	A multicenter retrospective analysis	300	PSMA PET/CT	Patients with biochemical recurrence and negative PSMA-PET scans still derive clear oncologic benefit from early sRT, supporting the guideline recommendation not to delay treatment solely due to a negative PET result.	Limitations include the retrospective design, nonstandardized treatment protocols across institutions, categorical (not continuous) recording of PSA levels, and imbalance in use of nodal RT or ADT.	Adebahr et al.^144^
2023	A single-center prospective study	75	[^18^F]-fluciclovine PET/CT	Pre-sRT 18F-fluciclovine PET/CT findings are predictive of sRT outcomes. A negative scan is strongly associated with improved FFS, while pelvic nodal involvement, especially in multiple nodes, indicates a high risk of early failure.	Small sample size limits generalizability and may obscure subtle differences in subgroups. Single-center design may introduce institutional bias and reduce external validity.	Lawal et al.^146^
2023	A prospective, randomized, phase 2/3 controlled trial (EMPIRE-1, NCT01666808)	165	[^18^F]-fluciclovine PET/CT	[^18^F]-fluciclovine PET/CT improves target delineation and patient selection, leading to improved treatment outcomes, especially in patients with lower PSA levels and favorable pathology.	Exploratory subgroup analyses; small sample size in certain subgroups (e.g., PSA ≥ 2 ng/mL); no comparison with newer PET tracers like PSMA PET/CT	Lawal et al.^147^
2024	A single-center prospective study	60	[^11^C]-choline PET/CT	ENRT with PET-guided boost provides durable disease control, particularly in HSPC patients, with acceptable toxicity. Median CRFS was 67 months and OS 110 months.	Limitations include the single-center, nonrandomized design; use of [^11^C]-choline PET instead of PSMA PET, which may have reduced diagnostic sensitivity; and the heterogeneity in systemic therapies used during follow-up.	Fodor et al.^148^
2025	A single-center retrospective analysis	89	[^68^Ga]Ga-PSMA-11 PET/CT	PSMA uptake in irradiated lesions decreased progressively, with the lowest SUVmax seen at 9–12 months postradiotherapy. Residual uptake was more frequent in prostate/prostate-bed lesions, those with high baseline SUVmax, and those imaged earlier after treatment (median 7.9 vs. 13.0 months, *p* = .001).	It is retrospective and single-center, with follow-up imaging performed mainly in cases with suspected recurrence, introducing selection bias.	Hotta et al.^149^
2024	A retrospective multicenter analysis	402	[^68^Ga]Ga-PSMA-11, [^18^F]F-PSMA-1007, [^18^F]-fluciclovine PET/CT	The type of PET tracer used to guide MDT significantly influenced long-term outcomes in oligorecurrent PCa. PSMA PET/CT, particularly with [^68^Ga]Ga-PSMA-11, led to longer PFS, delayed systemic therapy initiation, and improved OS compared to choline PET/CT.	The study’s retrospective nature and lack of centralized imaging review introduce potential biases, especially regarding interpretation variability for [^18^F]F-PSMA-1007.	Bauckneht et al.^150^
2023	A single-center prospective study	58	[^18^F]DCFPyL PET/CT	PSMA PET/CT-directed MDT in patients with biochemical recurrence, absent systemic therapy, resulted in substantial PSA responses and promising BPFS outcomes. These benefits were independent of ADT, supporting the role of image-guided local interventions.	The study is limited by its retrospective design, small sample size, and lack of a control group. Most patients were high risk at diagnosis, limiting generalizability.	Harsini et al.^151^
2024	A single-center prospective study	101	[^18^F]DCFPyL PET/CT	Even in the context of a negative PSMA PET/CT scan, early administration of sRT is associated with improved freedom from clinical progression in men with biochemical recurrence post-RP. Additionally, the ISUP grade at initial diagnosis—particularly grade 5—was a strong predictor of recurrence.	It is subject to selection bias, especially in the assignment of patients to surveillance versus treatment.	Harsini et al.^152^
2025	A multicenter retrospective analysis	82	[^68^Ga]Ga-PSMA-11 PET/CT	PSMA-PET/CT SUVmax is a valuable prognostic biomarker in mHSPC, particularly in predicting the efficacy of treatment with ADT + ARSI.	Limitations include its retrospective nature, relatively small sample size, and short median follow-up of 11.7 months.	Henríquez et al.^153^
2025	A multicenter prospective study	183	[^68^Ga]Ga-PSMA PET/CT	PSMA SUVmax is a strong, independent predictor of biochemical recurrence following curative treatment. Importantly, it challenges current risk stratification paradigms by demonstrating that high intraprostatic SUVmax can override the prognostic implications of low biopsy ISUP grades.	Limitations include a relatively short follow-up period (median 3.15 years), lack of uniform androgen-deprivation therapy data, and reliance on retrospective PSA data collection.	Ades et al.^154^
2024	A single-center retrospective analysis	71	[^68^Ga]Ga-PSMA PET/CT	RT significantly reduced PSMA uptake in the primary tumor across nearly all patients (93%), with a median SUVmax decrease of 61.2%. Patients receiving RT plus ADT had significantly greater reductions in both SUVmax (59.1% vs. 45.1%, *p* = .004) and PSA levels (92.7% vs. 55.7%, *p* < .001), as well as a higher complete metabolic response rate (40% vs. 0*%, p* < .001) compared to RT alone.	Limitations include the retrospective design, modest sample size (*n* = 71), and lack of long-term follow-up to correlate early imaging findings with clinical outcomes.	Onal et al.^157^
2023	A single-center retrospective analysis	71	[^68^Ga]Ga-PSMA-11-PET/CT	Metabolic response on [^68^Ga]Ga-PSMA-11 PET/CT after nADT is a valuable prognostic marker in high-risk PCa patients undergoing RT. Poor SUV response (SD/PD) was associated with higher risk of biochemical failure and worse bDFS, particularly in patients with high Gleason scores and lymph node metastases.	Absence of histological confirmation of treatment response and variability in SUV measurements due to prostate size reduction after ADT may affect result reliability.	Onal et al.^158^
2024	A retrospective multicenter analysis	248	[^68^Ga]Ga-PSMA PET/CT	Early clinical T stage and staging with PSMA PET/CT were independently associated with better PCSS. Definitive RT combined with ADT is an effective approach for node-positive PCa patients.	The study’s limitations include its retrospective design and selection bias due to nonrandom imaging allocation.	Onal et al.^159^

Abbreviations: ADT, androgen-deprivation therapy; ARSIs, androgen receptor signaling inhibitors; bDFS, biochemical disease-free survival; bFS, biochemical failure-free survival; bPFS, biochemical progression-free survival; BRFS, biochemical recurrence-free survival; CRFS, clinical recurrence-free survival; CTV, clinical target volume; dRT, definitive radiotherapy; EAU, European Association of Urology; EBRT, external beam radiotherapy; ENRT, extended nodal radiotherapy; FFS, failure-free survival; GTV, gross tumor volume; GTVn, gross tumor volume of nodal disease; HDR-BT, high-dose-rate brachytherapy; HPRT, hemi-pelvic radiotherapy; HSPC, hormone-sensitive; LN, lymph node; LT-ADT, long-term androgen-deprivation therapy; mCRPC, metastatic castration-resistant prostate cancer; MDT, metastasis-directed therapy; MFS, metastasis-free survival; MHRT, moderately hypofractionated radiotherapy; mHSPC, metastatic hormone-sensitive prostate cancer; mpMRI, multiparametric magnetic resonance imaging; MRgRT, magnetic resonance-guided radiotherapy; NPV, negative predictive value; OS, overall survival; PCa, prostate cancer; PCSS, PCa-specific survival; PET/CT, positron emission tomography/computed tomography; PIVOTAL, Prostate Cancer Expert Group-defined Pelvic Irradiation Volume and Treatment Outcome Trial; PSA, prostate-specific antigen; PSMA, prostate-specific membrane antigen; PTV, planning target volume; RT, radiotherapy; RTOG, Radiation Therapy Oncology Group; SABR, stereotactic ablative body radiotherapy; SIB, simultaneous-integrated boost; sRT, salvage radiotherapy; TE-FS, treatment escalation-free survival.

## Data Availability

Data will be made available upon request.
